# Use of Network Pharmacology and Molecular Docking Technology to Analyze the Mechanism of Action of Velvet Antler in the Treatment of Postmenopausal Osteoporosis

**DOI:** 10.1155/2021/7144529

**Published:** 2021-10-11

**Authors:** Kuiting Guo, Tiancheng Wang, Enjing Luo, Xiangyang Leng, Baojin Yao

**Affiliations:** ^1^Changchun University of Chinese Medicine, Changchun 130117, China; ^2^Jilin Ginseng Academy, Changchun University of Chinese Medicine, Changchun 130117, China

## Abstract

Deer velvet antlers are the young horns of male deer that are not ossified and densely overgrown. Velvet antler and its preparations have been widely used in the treatment of postmenopausal osteoporosis (PMOP) in recent years, although its mechanism of action in the human body remains unclear. To screen the effective ingredients and targets of velvet antler in the treatment of PMOP using network pharmacology and to explore the potential mechanisms of velvet antler action in such treatments, we screened the active ingredients and targets of velvet antler in the BATMAN-TCM database. We also screened the relevant targets of PMOP in the GeneCards and OMIM databases and then compared the targets at the intersection of both velvet antler and PMOP. We used Cytoscape 3.7.2 software to construct a network diagram of “disease-drug-components-targets” and a protein-protein interaction (PPI) network through the STRING database and screened out the core targets; the R language was then used to analyze the shared targets between antler and PMOP for GO-enrichment analysis and KEGG pathway-annotation analysis. Furthermore, we used the professional software Maestro 11.1 to verify the predictive analysis based on network pharmacology. Hematoxylin-eosin (H&E) staining and micro-CT were used to observe the changes in trabecular bone tissue, further confirming the results of network pharmacological analysis. The potentially effective components of velvet antler principally include 17*β*-E2, adenosine triphosphate, and oestrone. These components act on key target genes such as AKT1, IL6, MAPK3, TP53, EGFR, SRC, and TNF and regulate the PI3K/Akt-signaling and MAPK-signaling pathways. These molecules participate in a series of processes such as cellular differentiation, apoptosis, metabolism, and inflammation and can ultimately be used to treat PMOP; they reflect the overall regulation, network regulation, and protein interactions.

## 1. Introduction

Postmenopausal osteoporosis (PMOP) refers to a metabolic disease in which the endocrine function of women's ovaries declines after menopause and the levels of estrogen diminish, which in turn causes bone resorption by osteoclasts to exceed bone formation by osteoblasts [1–3]. PMOP is characterized by a progressive reduction in systemic bone density and trabecular and cortical bone thinning and changes in the microstructure of bone tissue [4], leading to clinical manifestations such as pain, shortened body length, hunchback, and fractures. With the increased aging of the general population, the incidence of PMOP has also escalated annually and seriously affected the quality of life of middle-aged and elderly women, thus greatly raising the overall medical burden to society. Therefore, the prevention and treatment of PMOP are particularly important. There have been many studies in recent years on the pathogenesis of PMOP, and they have shown that [5–12] numerous factors are closely related to the pathogenesis of PMOP, including reproductive history, age, dietary exercise, body mass index, and genetics. The clinical treatments for PMOP principally include bisphosphonates, hormone-replacement therapy, selective estrogen receptor modulators, and parathyroid hormone; however, the effects of those treatment methods usually cause obvious side effects [13].

Deer antler was included in the 2020 Chinese Pharmacopoeia [14], and its principal actions include warming the kidneys and strengthening yang, nourishing essence and blood, strengthening muscles and bones, regulating the conception vessel and thoroughfare vessel, and neutralizing harmful toxins. The Compendium of Materia Medica states that velvet antlers “produce the essence and nourish the marrow, nourish the blood and benefit the yang, and strengthen the muscles and bones” [15]. Researchers found that velvet antler contains velvet polypeptides, steroids, velvet polysaccharides, velvet polyamines, inorganic elements, growth factors, and other ingredients [16]. Kim reported that deer antlers promote longitudinal bone growth in growing rats by enhancing BMP-2 expression, osteogenic activity, and bone matrix gene expression [17]. Research by Tseng et al. showed that deer antlers prevent bone loss by promoting bone formation and reducing bone resorption [18]. Our group further demonstrated that the therapeutic effects of deer antler on treating bone diseases might be achieved through the regulation of bone formation and remodeling by controlling a series of serum proteins and signaling pathways that were essential for osteoblast and osteoclast activities [19]. Although velvet antler contains various known components, the material basis and mechanism of action for use in the treatment of PMOP are still unclear.

Network pharmacology involves biology, biological network construction and analysis, connectivity, redundancy, and pleiotropy. It is used to reveal the effectiveness, toxicity, and metabolic properties of drugs. Network pharmacology transcends the traditional drug research and development model dominated by “one drug, one target, and one disease” and opens a new research model of the complex network relationship among multiple targets and multiple diseases. Velvet antler and its preparations have been widely used in the treatment of PMOP in recent years, but its underlying mechanism of action in the human body is still unclear. Therefore, in the present study, we used network pharmacology to screen the primary active ingredients and key targets of velvet antler treatment for PMOP and explored the mechanism of action of velvet antler in a “multicomponent, multitarget, and multipathway” manner.

We herein screened the active ingredients and targets of velvet antler in the BATMAN-TCM database. We also screened the relevant targets of PMOP in the GeneCards and OMIM databases and then compared the targets between velvet antler and PMOP. The target points of osteoporosis were then taken as an intersection. We used Cytoscape 3.7.2 software to draw the network diagram of “disease-drug-component-target.” We constructed a protein-protein interaction network (PPI) through the STRING database and screened out core targets. The R programming language was adopted to perform GO-enrichment analysis and KEGG pathway-annotation analysis on common targets of velvet antler and PMOP. Eleven core targets were obtained from the PPI network for molecular docking analysis. We then utilized the experimental results to develop our conclusions.

## 2. Materials and Methods

### 2.1. Screening of Deer Antler Active Ingredients and Targets

In the BATMAN-TCM, a bioinformatics analysis tool platform for elucidating the molecular mechanisms underlying traditional Chinese medicine, “deer antler” was selected as the keyword and “drug-target” as the screening condition, with the similarity model threshold score cut-off ≥20 and *p* < 0.05. Thus, the active ingredients contained in deer antler and their corresponding potential targets were screened.

### 2.2. PMOP-Related Target Screening and Velvet Antler Treatment of PMOP and Potential Target Prediction

In the GeneCards and OMIM databases, “postmenopausal osteoporosis” was used as the search term for all gene targets related to PMOP and then the intersection of the selected PMOP targets and the potential targets of deer antler were assessed, that is, the potential targets of velvet antler in the treatment of PMOP.

### 2.3. Construction and Analysis of the “Disease-Drug-Component-Target” Regulatory Network

The intersection target data for PMOP and velvet antler were input into Cytoscape 3.7.2 software to construct the regulatory network “disease-drug-component-target.” The different network nodes, respectively, represented PMOP, the traditional Chinese medicine velvet antler, the effective components of velvet antler, and the common targets of PMOP and velvet antler; the connecting line between the nodes represented the mutual relationships and effects.

### 2.4. Construction of the PPI Network and Screening of Core Targets

The common targets of PMOP and velvet antler were input in the STRING database (http://string-db.org) and the screening condition was set—species “*Homo sapiens*”—with the remaining conditions set as defaults; thus, the PPI network for velvet antler in the treatment of PMOP was constructed. The Cytoscape 3.7.2 software plug-in network analyzer was used to analyze the topological parameters of the average and maximal degrees of freedom in the PPI network. The core targets were screened out based on the metric value. Those with more chance to be the core ones of the treatment had larger value. The upper limit of the screening range was the maximum degree value in topological data, and the lower limit was twice the average degree of freedom [20, 21].

### 2.5. GO Functional Enrichment Analysis and KEGG Pathway-Enrichment Analysis

The R programming language was used to perform GO-enrichment analysis and KEGG pathway-annotation analysis on the intersecting targets obtained in the above-mentioned process, with the threshold *p* < 0.05. Through the results output, the possible mechanism of velvet antler treatment of PMOP was analyzed.

### 2.6. Molecular Docking

The Maestro 11.0 software was utilized to build the molecular docking model to evaluate the importance of candidate targets in drug therapy. The target prediction for the molecular docking was principally divided into the following four steps. ① First, the preparation of the crystal structure of the protein. The crystal structure was all from the RCSB Protein Data Bank database. Those were imported into Maestro to add hydrogen atoms and reasonably remove water molecules and irrelevant ligands of the structure as well as add incomplete amino acid residues. The structure was then optimized for the conformation that minimized energy. ② Preparation of ligands in molecular docking. The ligand molecule was first imported into Maestro, the correct of all atoms and bond levels were checked, and then the structure was optimized to minimize energy as the candidate ligand for docking. ③ Establishment of binding pockets during molecular docking. In this step, the original ligand in the cocrystal of the receptor protein was removed, and the size of the binding pocket was established. ④ Docking the ligand. The XP method for docking was selected.

### 2.7. Verification of the Animal Experiment

#### 2.7.1. Drugs and Materials

The deer antler used in the following experiments was the same as the ones prepared as previously described [22]. Antler tissue was collected from three anesthetized 4-year-old Chinese sika deer at the Shuangyang Deer Farm in Changchun, China. Deer antlers were fully rinsed with cold MilliQ water and thoroughly homogenized using a high-speed tissue homogenizer (Voshin, China). The homogenate was centrifuged at 12,000 × g and 4°C for 30 min. The supernatant was filtered through a 0.45 *μ*m Hollow Fiber Cartridge (GE Healthcare, Chicago, IL). The filtrate was freeze-dried by a Heto PowerDry LL3000 Freeze Dryer (Thermo, Waltham, MA) and stored at −80°C. When the mice need to be administered by gavage, the velvet antler was dissolved in pure water. FA1004N electronic balance (Shanghai Precision Scientific Instrument Co., Ltd.), Leica RM2255 paraffin microtome (Leica, Germany), Leica EG1140 paraffin-embedding machine (Leica, Germany), Olympus BX51 optical microscope (Olympus, Japan), and NIS-ELEMNT BR image analysis system (Japan NIKON Company) were used.

#### 2.7.2. Animals and Models

Eight-week-old C57 female mice were purchased from Liaoning Changsheng Biotechnology Co., Ltd. (experimental animal production license number, SCXK (Liao) 2020-0001). After 1 week of adaptive feeding, a dorsal approach was used to remove both ovaries. Models were created and randomly allocated to the control group and the administration group. All mice were intraperitoneally injected with penicillin sodium (40,000 units/mouse/day) for three consecutive days after the operation and were bred adaptively for one week. The control group was administered pure water intragastrically (i.g.), and the administration group was administered 4 mg/10 g/d deer antler i.g. for three consecutive weeks. After the experiment, the mouse femurs were removed under anesthesia. All experimental procedures were carried out in accordance with national and international guidelines and regulations, and all experiments were approved by the Animal Ethics Committee of the Changchun University of Traditional Chinese Medicine (no. ccucm-2017-0015).

#### 2.7.3. Microcomputed Tomography (Micro-CT) Analysis

The femur from each animal was sampled and the soft tissue was cleaned off. The microarchitecture of the femur was analyzed using a Quantum GX Micro-CT imaging system (PerkinElmer, Hopkinton, MA, USA). Each sample was carefully positioned such that the orientation of the femoral shaft was as vertical as possible. The distal femur was scanned with the following settings: 90 kV, 80 *μ*A, and a 14 min scanning time [23]. Bone morphometric parameters of the femur were obtained, including the bone volume over the total volume (BV/TV), trabecular number (Tb.N), and trabecular thickness (Tb.Th) [24].

#### 2.7.4. H&E Staining and Bone Static Histomorphometric Analysis

The femoral samples were fixed in 4% paraformaldehyde for 48 h, followed by decalcification in ethylenediaminetetraacetic acid (EDTA), dehydration in alcohol, clearing in xylene, and finally embedding in paraffin for further tissue sections. A paraffin microtome (RM2255, Leica, Germany) was used to section the sample into 4 *μ*m thick slices that were stained with hematoxylin and eosin (H&E). Section images were acquired with an Olympus BX51 optical microscope (Olympus, Japan). The distribution of trabecular bone was observed. The static parameters, namely, osteoblast surface/bone surface (ObS/BS), osteoclast surface/bone surface (OcS/BS), eroded surface/bone surface (ES/BS), and osteoid volume/bone volume (OV/BV), were analyzed with a quantitative stereological method for histology known as the Weibel technique [25, 26]. The static histomorphometric indices were performed at the secondary spongiosa area, which was rich in trabecular bone. The selected region was located 1 mm from the lateral cortex and 3–7 mm from the lowest point of the growth plate [27].

### 2.8. Statistical Analysis

The Shapiro–Wilk normality test was used to determine the distribution of the data as the sample size was less than 100. All of the groups were found to be normally distributed. Thus, the data were analyzed by SPSS 25.0 software using parametric Student's *t*-test (^*∗*^*p* < 0.05; ^*∗∗*^*p* < 0.01) and expressed as the mean ± SD.

## 3. Results

### 3.1. Screening of Active Ingredients in Velvet Antler

In BATMAN-TCM, with “antler” as the keyword, the screening condition set to “drug-target,” the similarity model threshold score cut-off set to ≥20, and *p* < 0.05 with strict screening, a total of 14 potential velvet antler active molecules were retrieved. The effective chemical components, as presented in Table 1, include 17-*β*-estradiol, glucosamine, adenosine triphosphate, and oestrone.

### 3.2. Predictive Results of Velvet Antler's Potential Targets in the Treatment of PMOP

**Figure 1:**
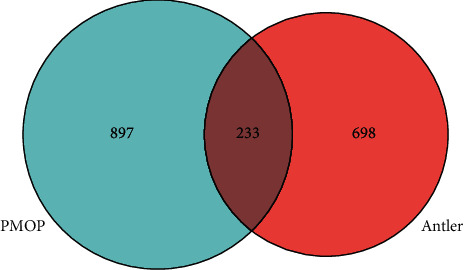
Venn diagram of velvet antler and postmenopausal osteoporosis (PMOP) targets. Light blue represents the PMOP targets, and red represents the velvet targets. The intersection of the two colors represents a total of 233 potential targets for velvet antler treatment of PMOP.

As shown in Figure 1, a total of 931 target points for active ingredients of velvet antler and 1,130 disease targets for PMOP were collected. We obtained an intersection between target points for velvet antler and PMOP and constructed a Venn diagram comprising a total of 233 intersection targets.

### 3.3. Results of the Construction of the Regulatory Network of “Disease-Chinese Medicine-Components-Targets”

**Figure 2:**
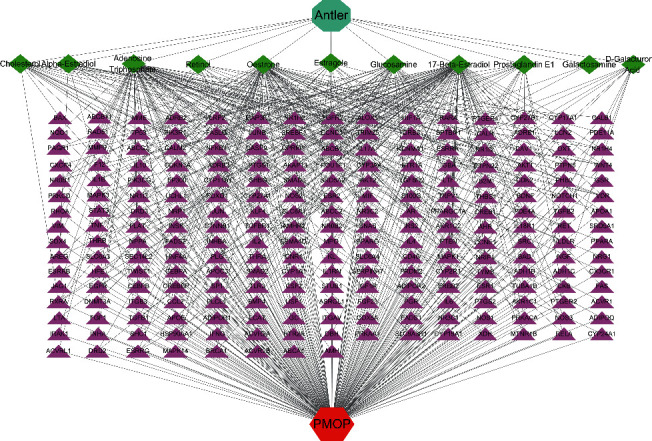
The “disease-drug-component-target” regulatory network of velvet antler in the treatment of postmenopausal osteoporosis. The triangle represents the target, the diamond represents the active ingredients of velvet antler, the regular octagon represents “velvet drugs,” and the hexagon represents postmenopausal osteoporotic disease. The straight lines connecting each node indicate the presence of regulatory relationship between the nodes.

The regulatory network comprising velvet antler for PMOP treatment is shown in Figure 2. In the figure, the regular octagon represents the traditional Chinese medicine velvet antler, the hexagon represents PMOP, the diamond represents the effective ingredients of velvet antler, and the triangle represents the common targets of the traditional Chinese medicine velvet antler and PMOP disease. Velvet antler primarily acts on 233 targets through 11 active ingredients (except for vitamin B1, lecithin, and 6′-O-*β*-D-glucosyl gentiopicroside), reflecting the multicomponent and multitarget effects of velvet antler on PMOP. As shown in Table 2, 17-*β*-estradiol, oestrone, and adenosine triphosphate exhibited the highest degree values, which are presumably the main active ingredients of velvet antler.

### 3.4. Analysis Results of the PPI Network

**Figure 3:**
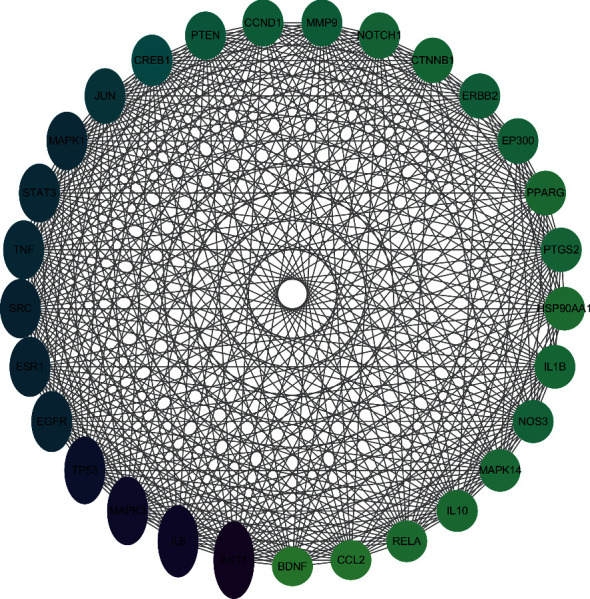
PPI network. The color and size of the nodes reflect the degree value. The larger and darker the nodes in the graph, the greater the DC value.

We entered 233 common targets in the STRING database to obtain protein interaction relationships and drew PPI network diagrams. The PPI network ultimately contained 230 nodes and 4434 edges. The network topology analysis results were as follows: network density (0.157), network heterogeneity (0.799), and shortest path (52670, 100%). According to the previous studies, the key core the lower limit should be twice the average degree of the nodes [20, 21]. Since the average degree of freedom of the nodes was 35.948, and the maximum freedom of degree was 142. Thus, there were 29 nodes with a greater-than-average degree, which should be considered as the key core targets. The 29 nodes were drawn with Cytoscape 3.7.2 according to the principle of “close to centrality”; the color of the node was positively correlated with the contribution of the node to the network. It can be seen from Figure 3 that the target proteins with high values close to the center include serine-threonine protein kinase (AKT1), interleukin-6 (IL-6), mitogen-activated protein kinase-3 (MAPK3), tumor suppressor P53 (TP53), epidermal growth factor (EGFR), estrogen receptor-1 (ESR1), proto-oncogene tyrosine-protein kinase Src (SRC), tumor necrosis factor (TNF), signal transducer and activator of transcription 3 (STAT3), mitogen-activated protein kinase 1 (MAPK1), and transcription factor AP-1 (JUN). These are the key core targets in the PPI network and thus may play an important role in velvet antler treatment of PMOP.

### 3.5. GO Functional Enrichment Analysis

**Figure 4:**
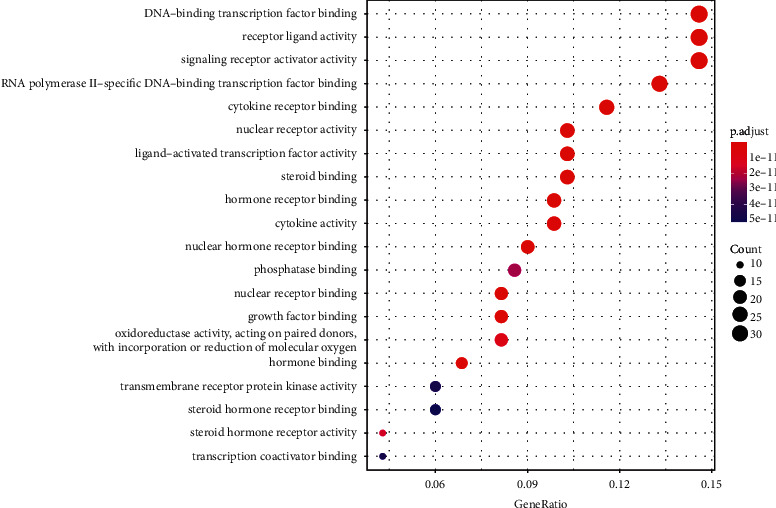
Analysis diagram of GO functional enrichment. The ordinate represents GO function items, the abscissa represents the proportion of genes, the size of the dots represents the number of enriched genes, and the color of the dots represents the range of different *P* values.

We obtained the GO functional enrichment analysis of the 233 potential targets, and the results are shown in Figure 4. It can be seen that GO functions principally involved DNA binding transcription factor binding, receptor ligand activity, signal transduction receptor activator, RNA polymerase II specific DNA binding transcription factor binding, cytokine receptor binding, and nuclear receptor activity. These results suggested that velvet antler treatment of PMOP includes the regulation of these biomolecules.

### 3.6. KEGG Pathway-Enrichment Analysis

**Figure 5:**
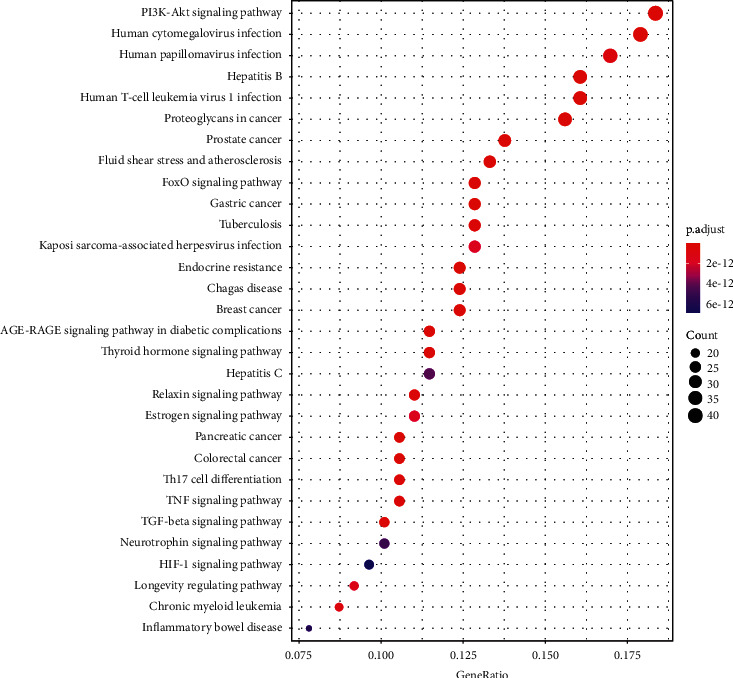
KEGG analysis diagram of key targets. The ordinate represents the name of the KEGG signal pathway, the abscissa represents the proportion of genes, the size of the dots represents the number of enriched genes, and the color of the dots represents the range of different *P* values.

**Figure 6:**
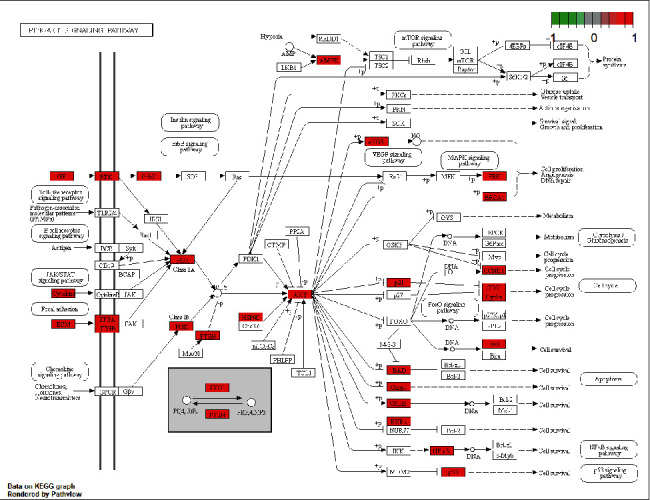
KEGG- and PI3K/Akt-signaling pathway: □ represents gene products; ○ represents compounds; red represents the key targets of deer antler; ⟶ represents intermolecular interactions or relationships.

The 233 potential targets of velvet antler treatment of PMOP were enriched in 178 signaling pathways (shown in Figure 5). It can be seen that the signaling pathways are primarily concentrated in the PI3K/Akt-signaling pathway, human cytomegalovirus infection, human papillomavirus, and so on. Thus, deer antler may exert their effects on PMOP by regulating these signaling pathways. The PI3K/Akt-signaling pathway was the most abundant pathway for potential target enrichment and is shown in Figure 6.

### 3.7. Molecular Docking Verification

**Figure 7:**
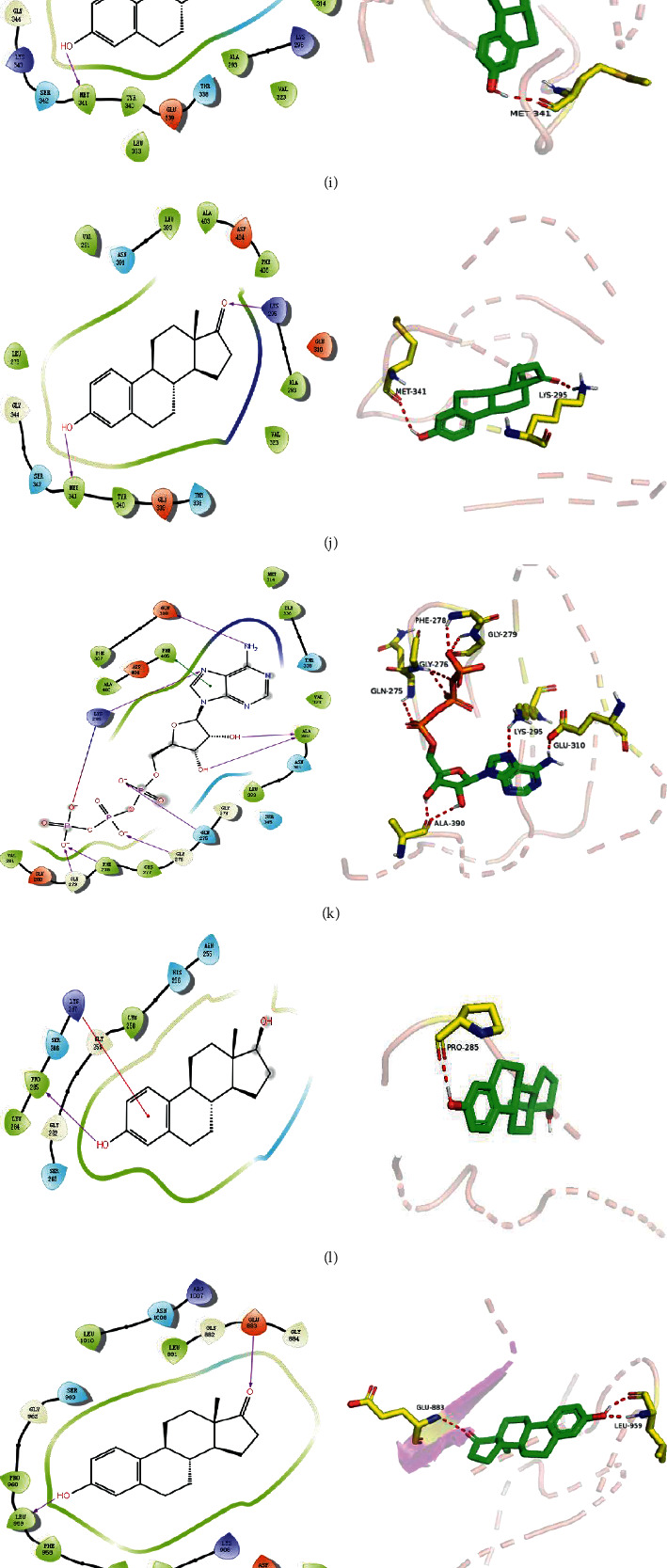
The molecular docking model of the top three active ingredients in velvet antler and the eleven core targets. In the 3D diagram, the red dotted line indicates the hydrogen bonds and related residues connected to each other, and the text represents the name of the residue. (a) AKT1 and 17-beta-estradiol; (b) AKT1 and oestrone; (c) AKT1 and adenosine triphosphate; (d) IL6 and adenosine triphosphate; (e) EGFR and 17-beta-estradiol; (f) EGFR and oestrone; (g) ESR1 and 17-beta-estradiol; (h) ESR1 and oestrone; (i) SRC and 17-beta-estradiol; (j) SRC and oestrone; (k) SRC and adenosine triphosphate; (l) STAT3 and 17-beta-estradiol; (m) STAT3 and oestrone; (n) STAT3 and adenosine triphosphate; (o) TNF and adenosine triphosphate; (p) MAPK1 and 17-beta-estradiol; (q) MAPK1 and oestrone; (r) MAPK1 and adenosine triphosphate; (s) JUN and 17-beta-estradiol; (t) JUN and oestrone; (u) JUN and adenosine triphosphate.

We selected eleven core target genes with a support degree higher than 100 for molecular docking and analyzed the docking results, with Gscore used in the scoring of molecular docking; the lower the score, the stronger the binding force. A negative value indicated that the ligand molecule could spontaneously bind to the receptor protein, and a score <−5 indicated stronger binding activity. A total of three compounds were selected to be docked on eleven target proteins, and the molecular docking scores of the compounds are summarized in Table 3. Finally, Figure 7 illustrates the compounds 17-*β*-estradiol, oestrone, and adenosine triphosphate, which bind to key targets and exhibit scores <−5. The visual results of the docking of other targets are shown in Figure 1 in the supporting materials.

### 3.8. Micro-CT Analysis

**Figure 8:**
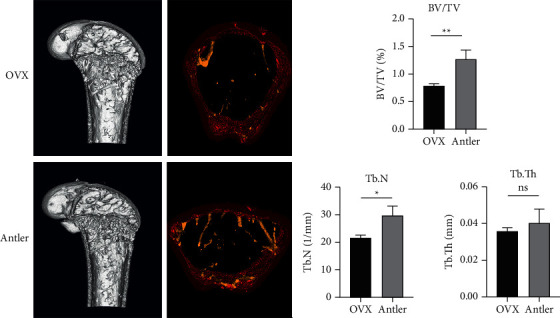
Micro-CT images and morphometric parameters in the distal femur. ^*∗*^*p* < 0.05; ^*∗∗*^*p* < 0.01.

To estimate the effect of deer antler treatment on trabecular bone structure, trabecular architectural parameters were measured in the distal femoral bone by using micro-CT analysis (Figure 8). The BV/TV and Tb.N in the deer antler groups significantly increased compared with those in the OVX group. However, Tb.Th in the deer antler group did not differ from that in the OVX group. The BV/TV ratio is an important parameter in the evaluation of the microstructure of the trabecular bone [28]. A lower BV/TV ratio is associated with fewer trabecular bones and morphological features. Antler treatment restored trabecular connectivity by increasing Tb.N and BV/TV ratio. These results indicate that deer antler can attenuate estrogen deficiency-induced bone loss, suggesting that deer antler might be a useful candidate for the treatment of osteoporosis in postmenopausal women.

### 3.9. H&E Staining Assessment

**Figure 9:**
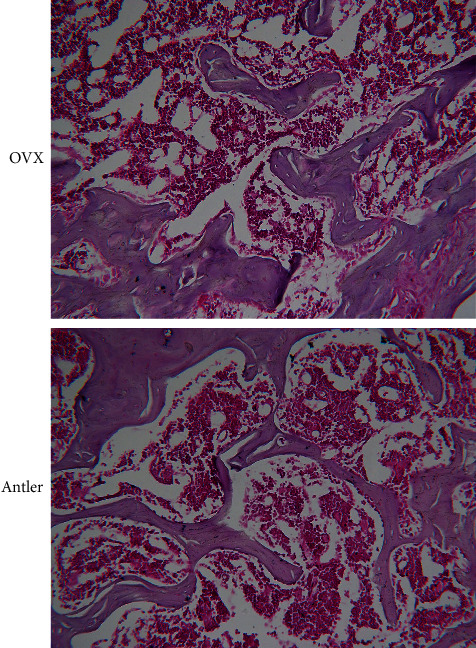
Hematoxylin and eosin staining of the distal femur, magnification ×200.

In the model group, the femoral shaft trabeculae were loosely distributed, the number of bone trabeculae was sparse, and the bone trabeculae showed low interconnectedness. The trabecular bone was thinner and thick locally. The distance between trabecular bones was augmented, and the proportion of the trabecular bone distribution area in the observation field was small. Compared with the model group, the number of trabecular bones in the femoral shaft of the deer antler group increased, and they were interconnected in a larger grid. The trabecular bones were locally thickened between the trabecular bones with the distance diminished. The trabecular bone distribution area accounted for the increase in the proportion of the observation field (see Figure 9).

### 3.10. Static Histomorphometry Parameters

**Figure 10:**
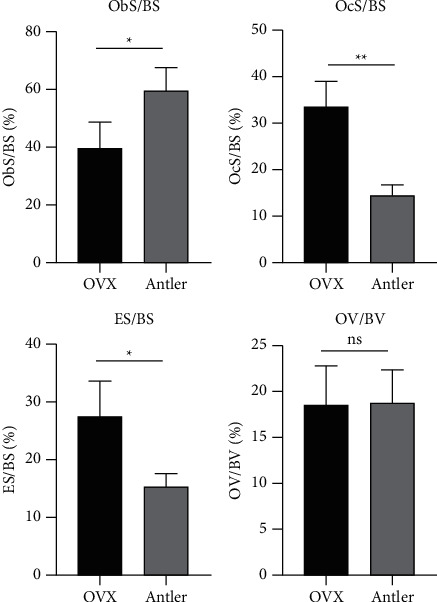
Static trabecular histomorphometry parameters after 3 weeks of antler treatment (^*∗*^*p* < 0.05; ^*∗∗*^*p* < 0.01).

According to the quantification analysis from the H&E staining, supplementation of antler significantly increased the ratio of ObS/BS and significantly decreased the ratios OcS/BS and ES/BS, compared to those in the OVX group. There was no significant difference in the ratio change of OV/BV after three weeks of antler treatment (Figure 10). These results indicated that antler supplementation could maintain better bone histomorphometric parameters, which enhances the possible role of deer antler in promoting bone formation and growth.

## 4. Discussion

Deer antler has been reported to possess bone-strengthening activity and has been used in treating arthritis, osteoporosis, and avascular necrosis of the femoral head in animal models, and it plays pivotal roles in facilitation of proliferation and mineralization of osteoblasts. Wang et al. reported that deer antler type I collagen could induce BMSC differentiation into osteoblasts through the two signal transduction pathways, ERK1/2 and p38-MAPK, and can regulate the gene expression of Runx2 and Osx, which were specific transcription factors in this case [29]. Yun et al. reported that deer antler peptide promoted osteoblast proliferation, differentiation, and mineralization in vitro via the insulin signaling pathway. The effect of deer antler peptide on insulin signaling in osteoblasts may be mediated via the ERK pathway and partially by the PI3K/Akt pathway [30]. Chen et al. reported that velvet antler dietary supplementation has beneficial effects on femur development in growing rats [31]. Shi et al. reported that the development of necrotic femoral head was prevented and replaced by regenerated tissue upon administration of dexamethasone-induced rats with antler extract. Deer antler extract (DAE) has a positive curative effect on avascular necrosis of the femoral head by promoting osteoblast proliferation [32]. On the other hand, there are also reports about the inhibitory effect of antler on osteoclast differentiation. Li et al. reported that DAE suppressed osteoclast differentiation by inhibiting phosphorylation of Akt, ERK, and I-*κ*B in response to RANKL. DAE also inhibited bone resorption and survival of osteoclasts [33]. In addition, our group has demonstrated that DAE significantly increased the expression levels of differentially expressed genes (DEGs) involved in cartilage growth and regeneration but decreased the expression levels of DEGs involved in inflammation and mildly increased the expression levels of DEGs involved in chondrogenesis and chondrocyte proliferation [34]. Thus, those findings indicate that deer antler extract has potential bone-protecting effects and might be beneficial for bone health.

In the present study, we initially collected the effective ingredients of velvet antler and screened them. We then obtained genes that are differentially expressed in PMOP from the GEO database and OMIM database, constructed a drug-component-target-disease network, and further concluded that 17*β*-E2, adenosine triphosphate, and oestrone were important components in velvet antler for use in the treatment of PMOP, the primary component of which is cytoporosis. Lack of estrogen is the principal cause of PMOP, and estrogen replacement therapy can effectively improve the bone density of postmenopausal women with osteoporosis. Ge et al. [35] demonstrated that estrogen inhibits the mTOR-signaling pathway by activating ERK, which then promotes chondrocyte autophagy and thereby protects articular cartilage. Estradiol regulates the maturation of osteoblasts under physiological conditions and prevents osteoblasts from undergoing apoptosis under stress conditions. Estrogen enhances the osteogenic activity of bone marrow mesenchymal stem cells through ER*α* activity [36] and can significantly reduce the expression of apoptosis-marker proteins in serum osteoblasts while increasing the expression of autophagy-marker proteins. Estradiol promotes autophagy through the ER/ERK/mTOR pathway in order to avoid osteoblast apoptosis [37] and promotes osteoblast differentiation by up-regulating bone morphogenetic protein-4 signaling [38]. ATP is a ubiquitous chemical-energy carrier and a component of all biological genetic material. A majority of the cell's ATP is in the mitochondria, and its generation primarily depends upon the coordinated expression of genes in the nucleus and mitochondria. As a signaling molecule outside the cell, ATP is involved in cell death [39]. Miyazaki et al. showed that extracellular ATP can also inhibit bone resorption by osteoclasts through purine receptors [40].

We constructed KEGG and PPI networks based on target information and determined twenty-nine core targets based on their degree values. AKT1, IL6, MAPK3, TP53, EGFR, ESR1, SRC, TNF, STAT3, MAPK1, and JUN were the key target genes with higher-node degrees; these genes can be considered to be the core targets in the PPI network and may thus be the key target genes for velvet antler treatment of PMOP. AKT1 belongs to the protein kinase B gene family and is a key factor in the PI3K/AKT-signaling pathway; it can phosphorylate elements downstream to regulate cellular metabolism, proliferation, survival, growth, and many other processes. Researchers have demonstrated that after knocking out the rat AKT1 gene, rats showed limited bone development, indicating that AKTI may promote bone growth in bone development [41]. Akt signaling in regulating bone metabolism is achieved by activating the downstream mTORC1/S6K1 protein, promoting osteoblast differentiation, and inhibiting osteoblast apoptosis [42]. Researchers [43–45] found that AKT1 is closely related to the proliferation and differentiation of osteoblasts and the formation of osteoclasts and can be used as a target in the treatment of osteoporosis. A significant feature of PMOP is chronic inflammation, and the increase in inflammatory factors such as tumor necrosis factor (TNF) and IL-6 can augment the expression of RANKL [46]. IL-6 affects bone loss and osteoporosis [47] and plays an important role in bone reconstruction [48]. MAPK3 (also known as ERK1) is a member of the MAPK-signaling pathway; studies have found that activated MAPK can reduce the differentiation of osteoclasts, promote the generation of osteoblasts, and improve osteoporosis [49]. Other studies [50] have shown that the hMSCs of patients with osteoporosis show a significant increase in the expression of TP53; moreover, TP53, SP1, and CTNNB1 transcription factors modulate the majority of the up-regulated differentially expressed genes. The above data also showed that the target protein screened by the PPI network was closely related to PMOP.

In this study, we used GO-enrichment and KEGG pathway-enrichment analyses to explore the multidimensional pharmacological mechanisms underlying the effects of velvet antler on PMOP. We also analyzed pathways containing other annotated genes, for example, viral infections with human cytomegalovirus, human papillomavirus, hepatitis B, human T-cell leukemia virus 1, and Kaposi sarcoma-associated herpesvirus. Based on this, we infer that velvet antler may have a broad-spectrum antiviral effect; and proteoglycans in cancer, prostate cancer, microRNAs in cancer, gastric cancer, breast cancer, and so on are mainly related to cancer. These approaches are not closely related to the treatment of postmenopausal osteoporosis with velvet antler. The PI3K/Akt- and MAPK-signaling pathways are two pathways that must be given more attention. The PI3K/AKT-signaling pathway is a key pathway for cellular proliferation and migration, and it is also a critical element in the development of osteoporosis. Overexpression of MIR-29b enhanced the proliferation and migration of bone marrow mesenchymal stem cells in ovariectomized osteoporotic rats, which may be due to the activation of PI3K/Akt- and TGF-*β*/Smad-signaling pathways [51]. The MAPK-signaling pathway regulates osteoblast apoptosis through the mitochondrial pathway [52], and there are currently three major MAPK signal transduction pathways: the ERK MAPK pathway, P38-MAPK pathway, and JNK MAPK pathway. Studies have shown that [53, 54] *Achyranthes bidentata* and *Eclipta prostrata* can regulate the ERK MAPK- and JNK MAPK-signaling pathways, respectively, to improve the former as major biomarkers of osteoblasts, and their augmented expression levels in turn promote the differentiation of BMSCs into osteoblasts.

We then used molecular docking technology to verify the combination of eleven core targets and three primary components of velvet antler; our results show that the molecular docking score Gscore of the three main components and the core target is negative, indicating that the ligand molecule can spontaneously bind to the receptor protein, and the score <−5 indicates that the binding activity is stronger.

Finally, in order to further confirm the results of the network pharmacology analysis, experimental verification was carried out using a postmenopausal osteoporotic mouse model. The structural and static histomorphometry parameters measure the microstructure and cellular components of the trabecular bone, respectively [55]. Micro-CT and H&E staining revealed that the trabecular bone levels of the distal femur of mice increased after gavage with velvet antler. It confirmed the reasonableness of network pharmacology analysis and molecular docking experiments.

## 5. Conclusions

In this study, we used a combination of network pharmacology and molecular docking to explore the mechanism of action of velvet antler in the treatment of PMOP. The potentially effective components of velvet antler primarily included 17*β*-E2, adenosine triphosphate, and oestrone. These components act on key target genes, such as AKT1, IL6, MAPK3, TP53, EGFR, ESR1, SRC, TNF, STAT3, MAPK1, and JUN, and regulate the PI3K/Akt- and MAPK-signaling pathways. These molecules and pathways then participate in a series of processes, such as cellular differentiation, apoptosis, metabolism, and inflammation, and can ultimately be used to treat PMOP, reflecting the overall regulation, network regulation, and protein interactions. This study can provide a certain theoretical basis for the research and development of related drugs for the clinical treatment of PMOP. However, the regulation mechanism of its action still needs to be further verified through experiments.

## Figures and Tables

**Table 1:** Potentially active ingredients in deer antler.

Compound	Molecular formula	PubChem CID	Molecular structure
17-Beta-estradiol	C_18_H_24_O_2_	5757	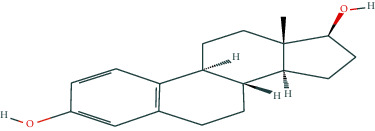

Glucosamine	C_6_H_13_NO_5_	439213	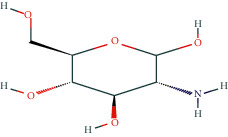

Adenosine triphosphate	C_10_H_16_N_5_O_13_P_3_	5957	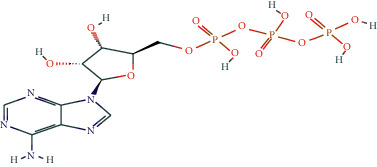

Estragole	C_10_H_12_O	8815	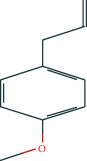

Alpha-estradiol	C_18_H_24_O_2_	68570	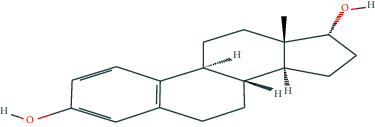

Retinol	C_20_H_30_O	445354	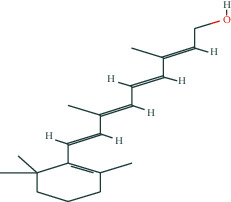

Lecithin	C_44_H_78_I_10_NO_8_P	161844	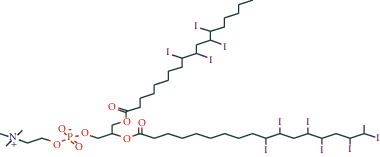

Galactosamine	C_6_H_13_NO_5_	24154	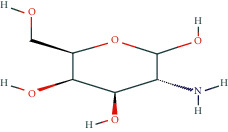

Cholesterol	C_27_H_46_O	5997	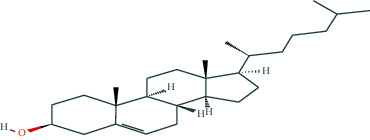

D-Galacturonic acid	C_6_H_10_O_7_	439215	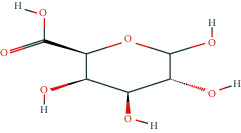

6′-O-Beta-D-glucosyl gentiopicroside	C_22_H_30_O_14_	10864232	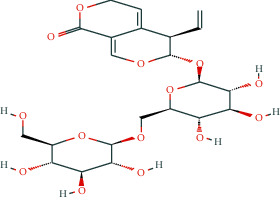

Prostaglandin E1	C_20_H_34_O_5_	5280723	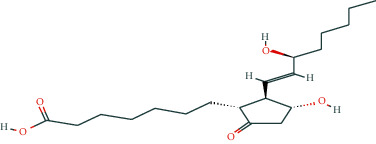

Vitamin B1	C_12_H_17_C_l_N_4_OS	6042	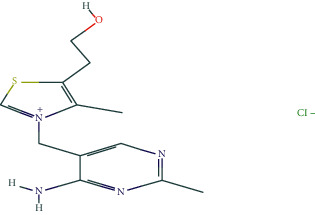

Oestrone	C_18_H_22_O_2_	5870	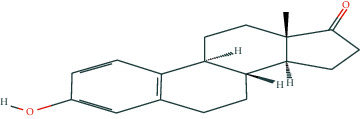

**Table 2:** Network topology properties of active ingredients.

Active ingredients	Betweenness centrality	Closeness centrality	Degree
17-Beta-estradiol	0.07184913	0.46934866	101
Oestrone	0.04359714	0.44064748	84
Adenosine triphosphate	0.05412014	0.43133803	78
Glucosamine	0.01278766	0.37692308	37
Cholesterol	0.00337715	0.35714286	19
Prostaglandin E1	0.00254506	0.35404624	16
Estragole	0.002092	0.35302594	15
Alpha-estradiol	0.00106778	0.35201149	14
Retinol	0.00149386	0.35201149	14
D-Galacturonic acid	0.00125548	0.34900285	11
Galactosamine	9.76*E−*05	0.34122563	3

**Table 3:** Molecular docking scores of compounds.

Core target	PDB ID	Active ingredients		
		17-Beta-estradiol	Oestrone	Adenosine triphosphate
AKT1	3OCB	−6.2	−6.2	−7.2
IL6	4CNI	−2.7	−2.3	−6.2
MAPK3	6GES	−3.2	−2.7	−4.7
TP53	6VIP	−2.8	−2.8	−4.0
EGFR	5UGB	−6.7	−6.1	−5.0
ESR1	5FQR	−10.5	−10.2	−2.5
SRC	3EL8	−6.8	−5.9	−6.0
STAT3	6SMB	−7.5	−7.5	−6.2
TNF	2AZ5	−3.1	−3.2	−5.0
MAPK1	6OPH	−-7	−6.9	−9.9
JUN	2P33	−7.2	−7.6	−13.2

## Data Availability

The research data used to support the findings of this study are included within the article.

## Supplementary Materials

Supplementary Materials Supplementary Figure 1: the molecular docking model of the top three active ingredients in velvet antler and the eleven core targets.
