# Supplementary material for: Use of Network Pharmacology and Molecular Docking Technology to Analyze the Mechanism of Action of Velvet Antler in the Treatment of Postmenopausal Osteoporosis
Source: Evid Based Complement Alternat Med. 2021 Oct 11;2021:7144529. doi: 10.1155/2021/7144529 (PMC8523247; doi:10.1155/2021/7144529)
Supplement: Supplementary Materials — Supplementary Figure 1: the molecular docking model of the top three active ingredients in velvet antler and the eleven core targets. [file 7144529.f1.doc]

## Supplementary Materials

Figure 1 The molecular docking model of the top three active ingredients in velvet antler and the eleven core targets.


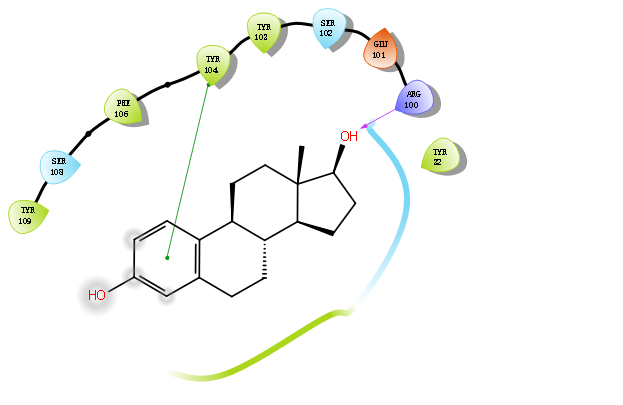

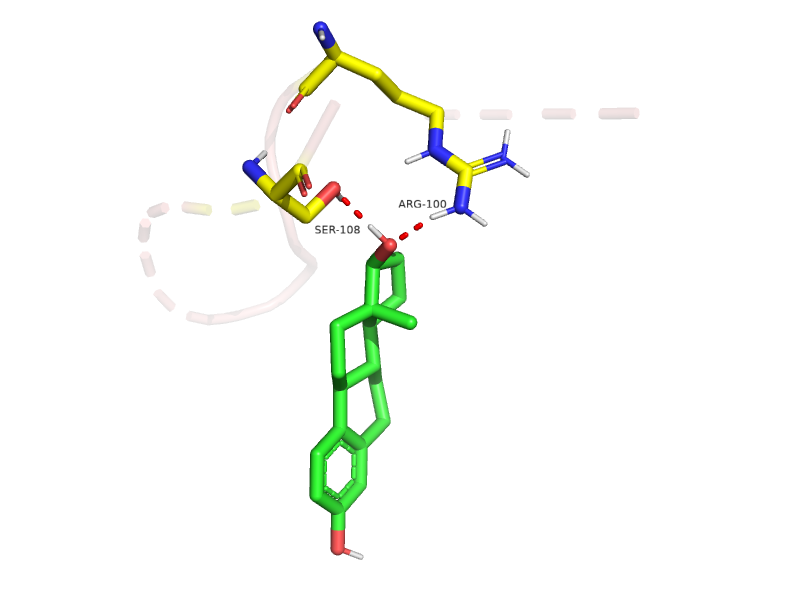


IL6 and [17-Beta-Estradiol](http://www.megabionet.org/tcmid/ingredient/23197/)


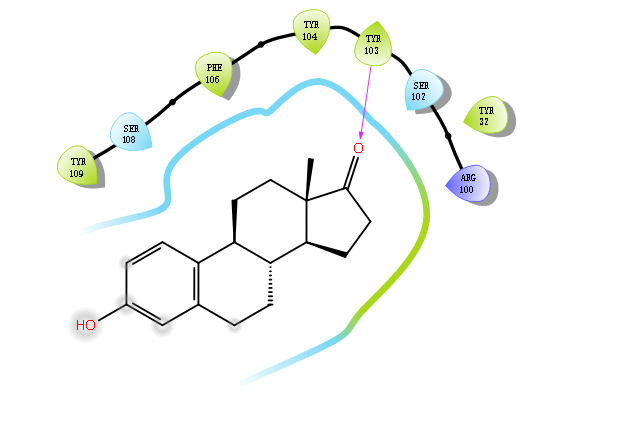

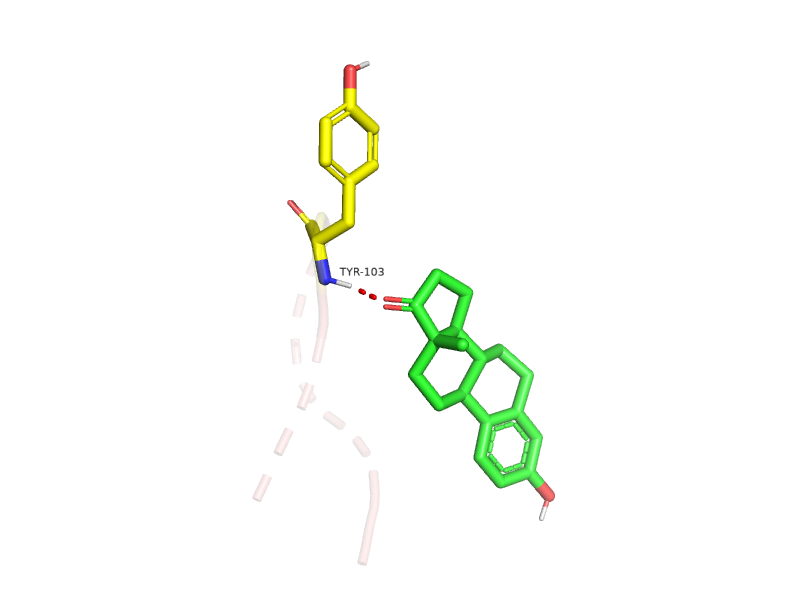


IL6 and [Oestrone](http://www.megabionet.org/tcmid/ingredient/23428/)


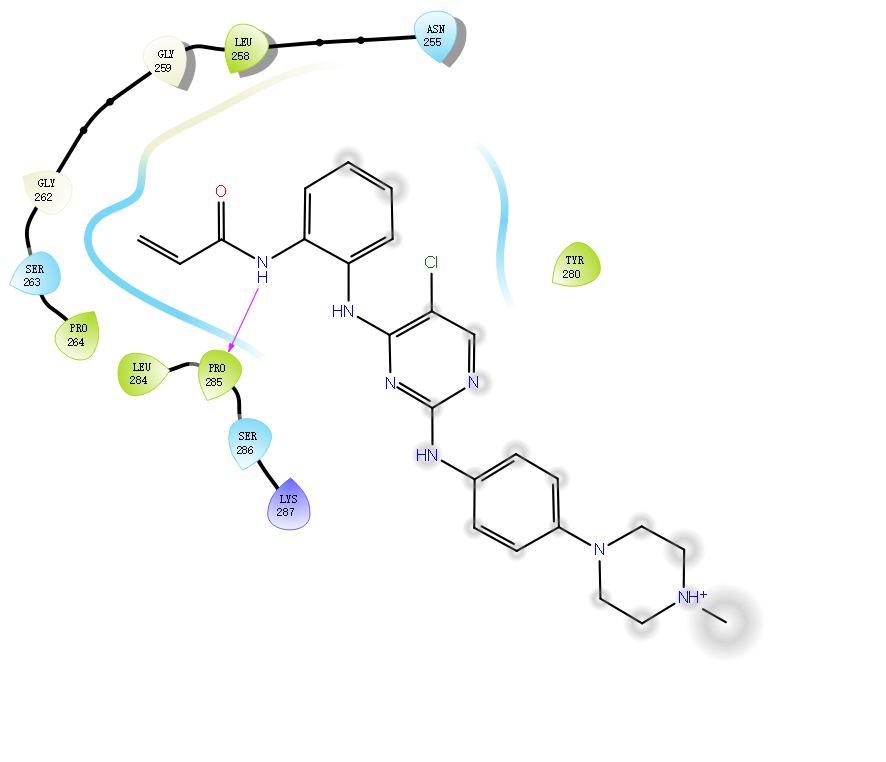

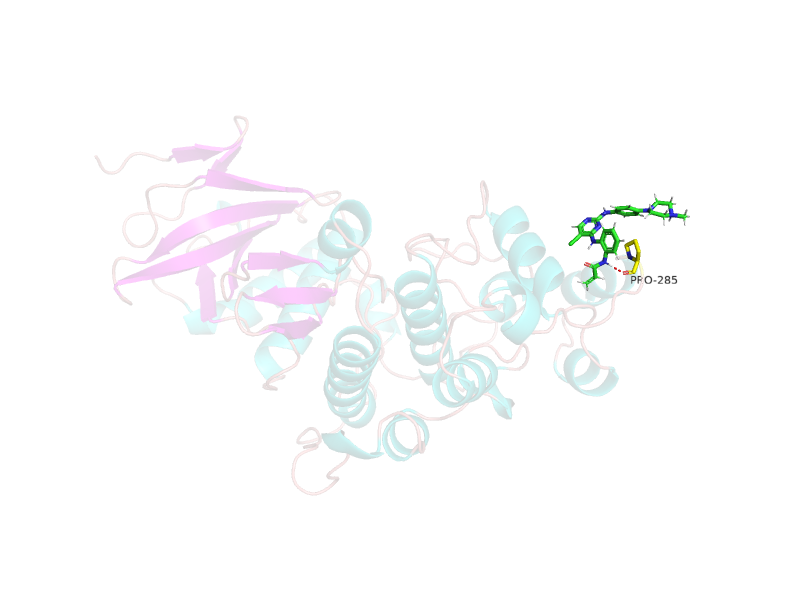


MAPK3 and [17-Beta-Estradiol](http://www.megabionet.org/tcmid/ingredient/23197/)


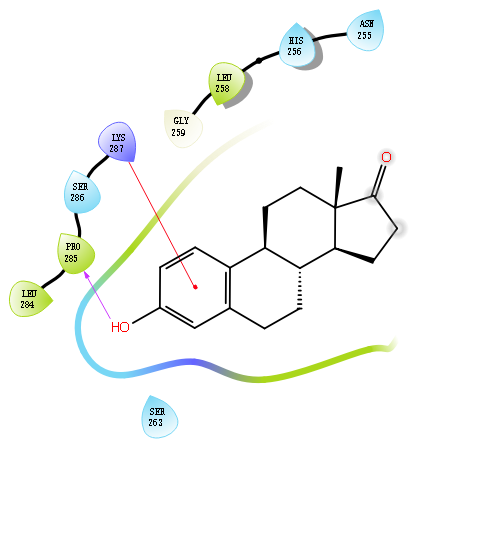

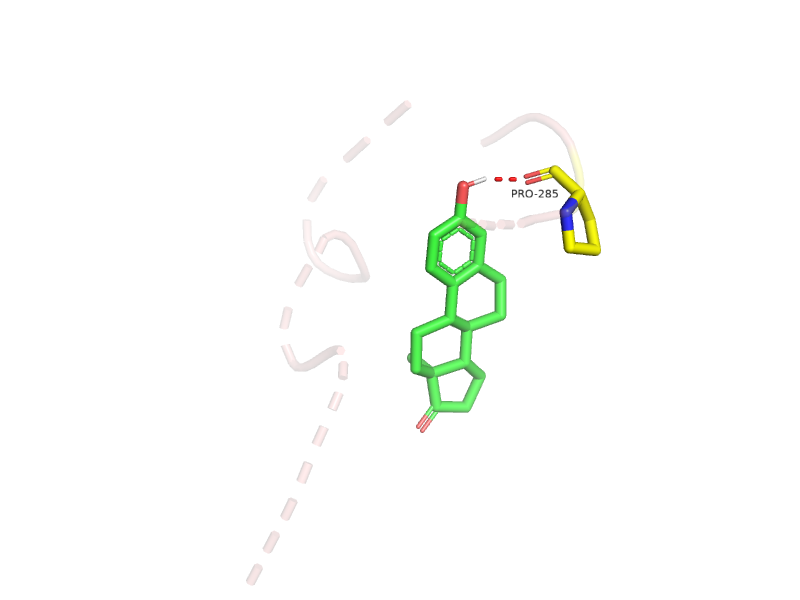


MAPK3 and [Oestrone](http://www.megabionet.org/tcmid/ingredient/23428/)


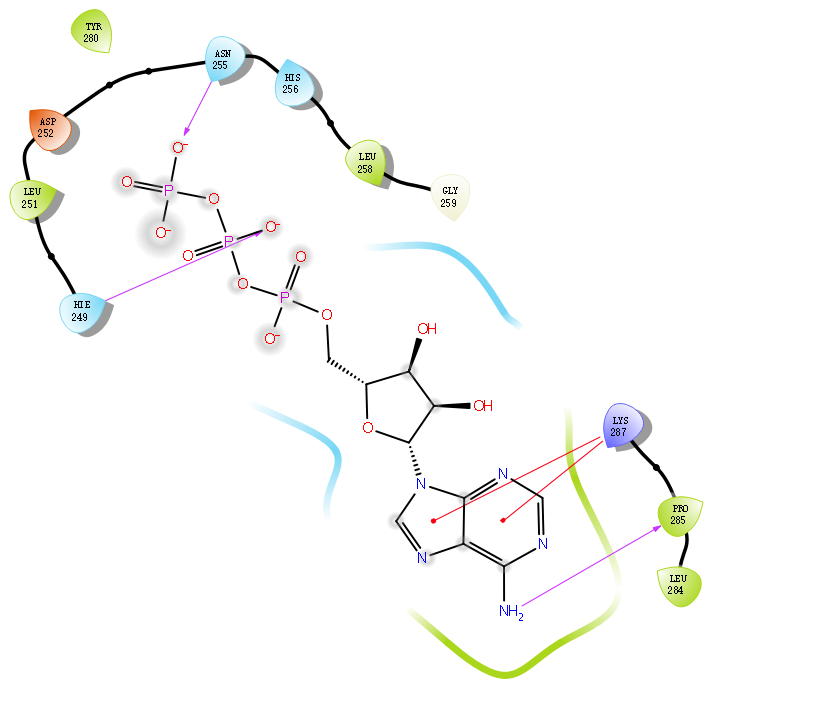

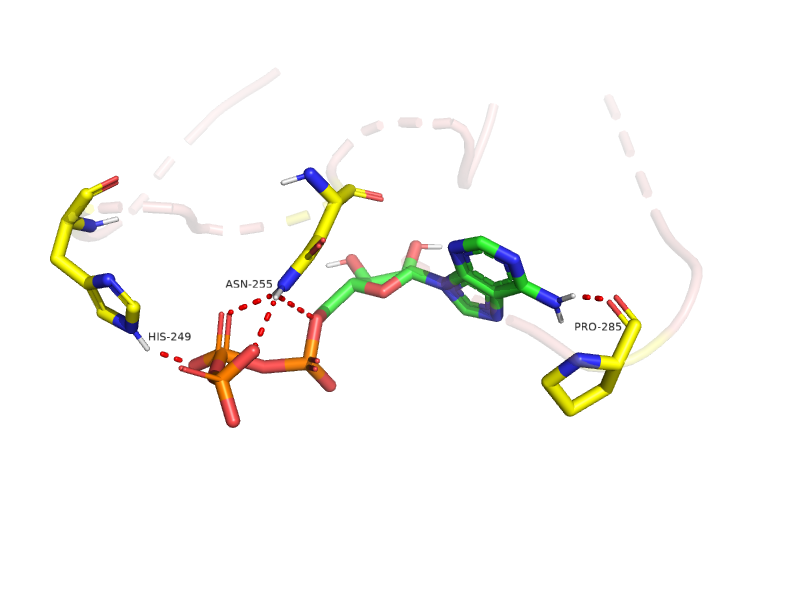


MAPK3 and [Adenosine Triphosphate](http://www.megabionet.org/tcmid/ingredient/626/)


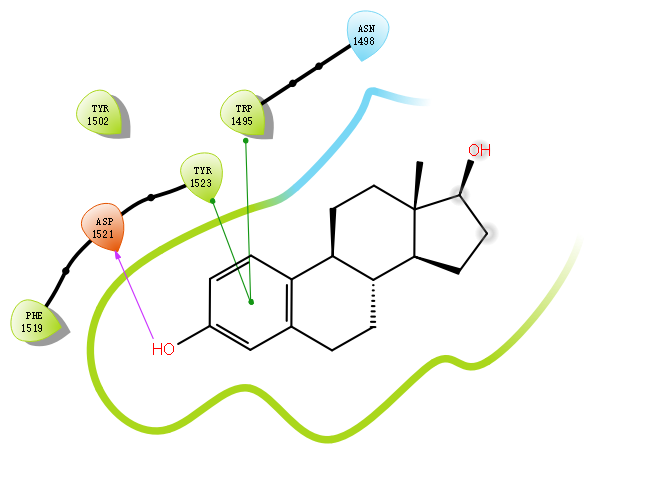

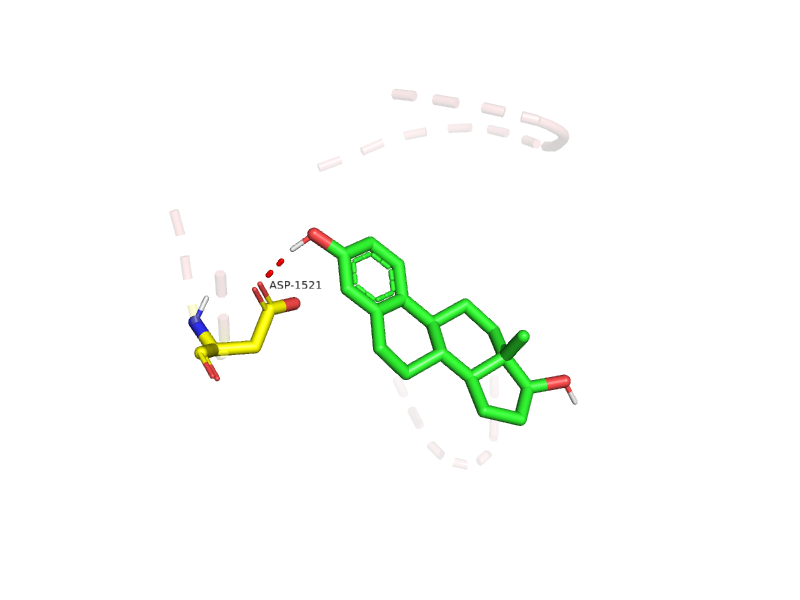


TP53 and [17-Beta-Estradiol](http://www.megabionet.org/tcmid/ingredient/23197/)


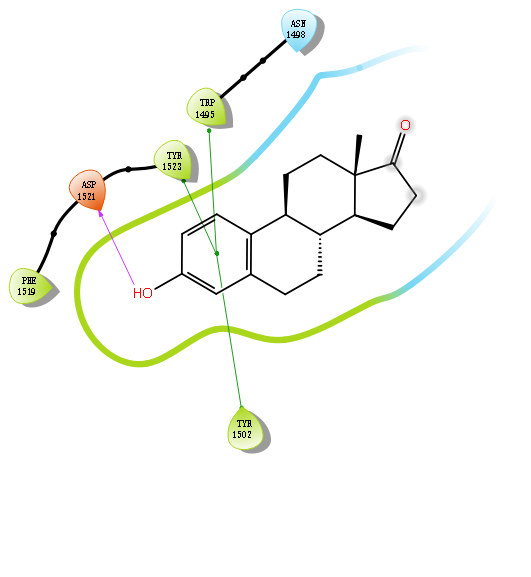

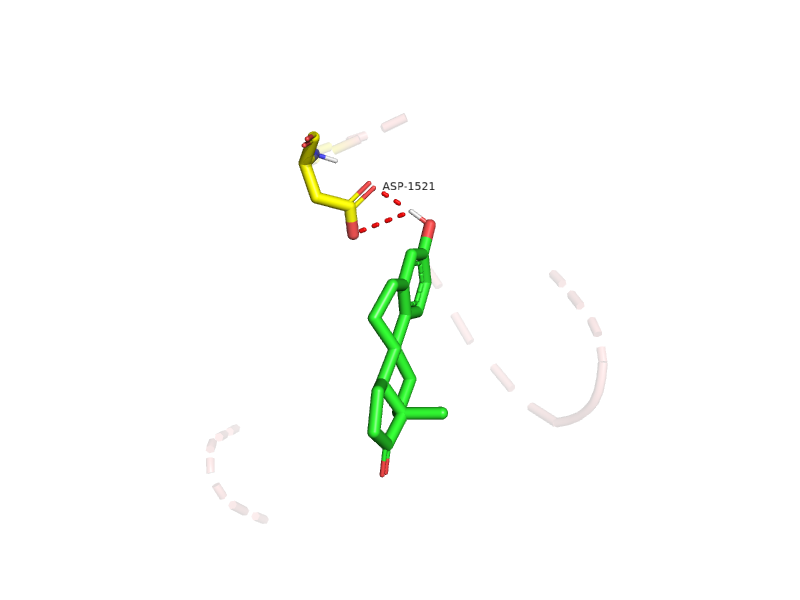


TP53 and [Oestrone](http://www.megabionet.org/tcmid/ingredient/23428/)


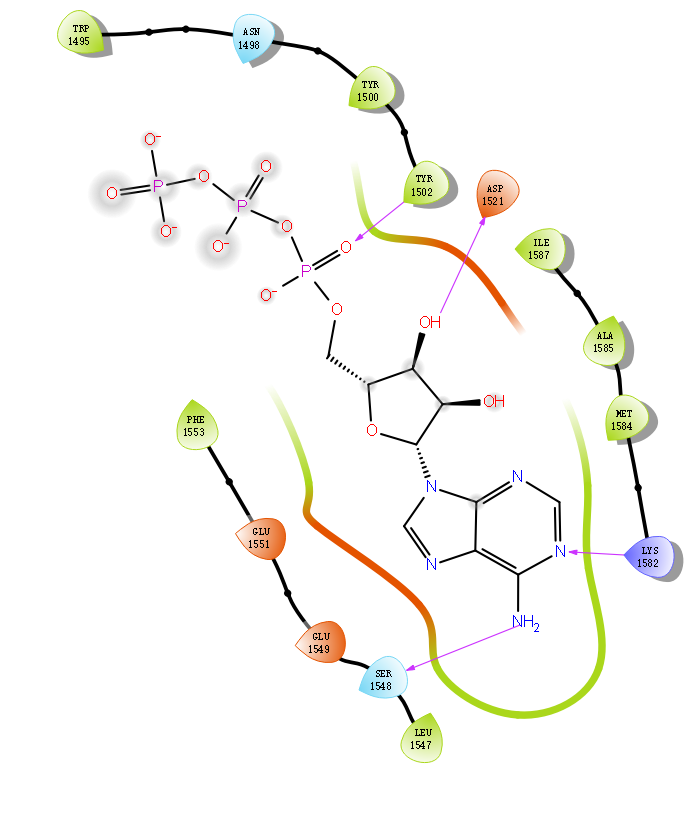

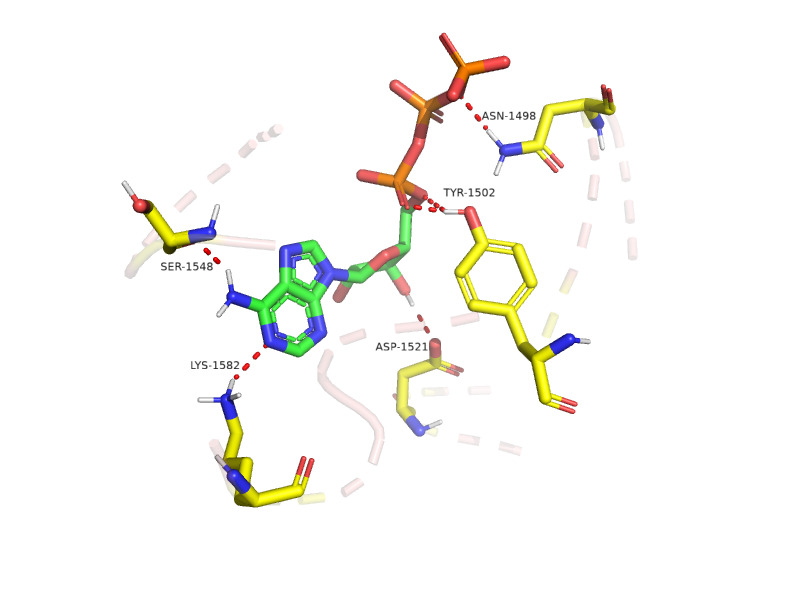


TP53 and [Adenosine Triphosphate](http://www.megabionet.org/tcmid/ingredient/626/)


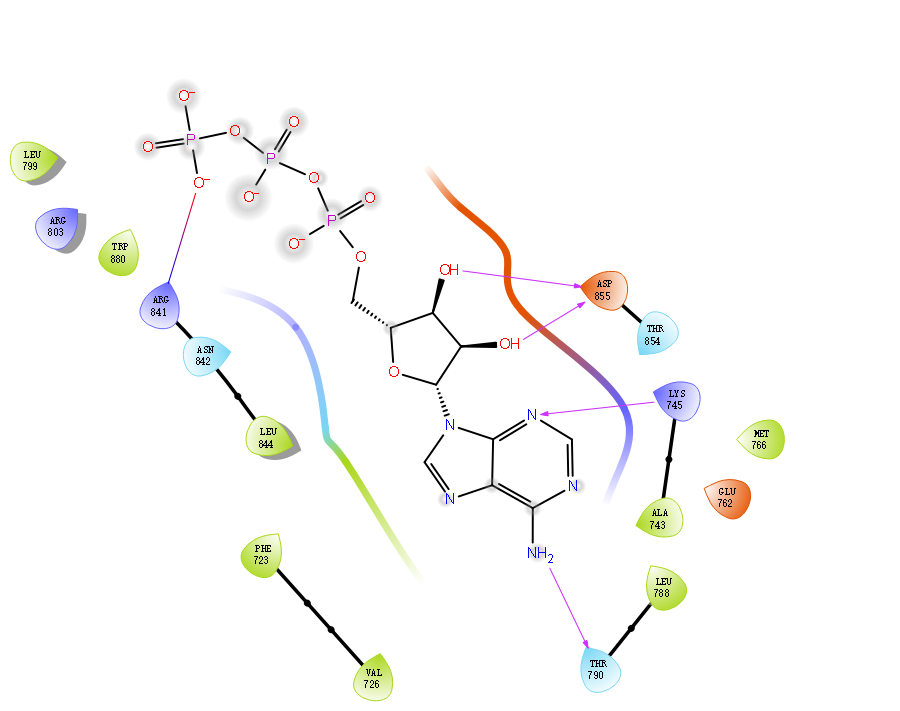

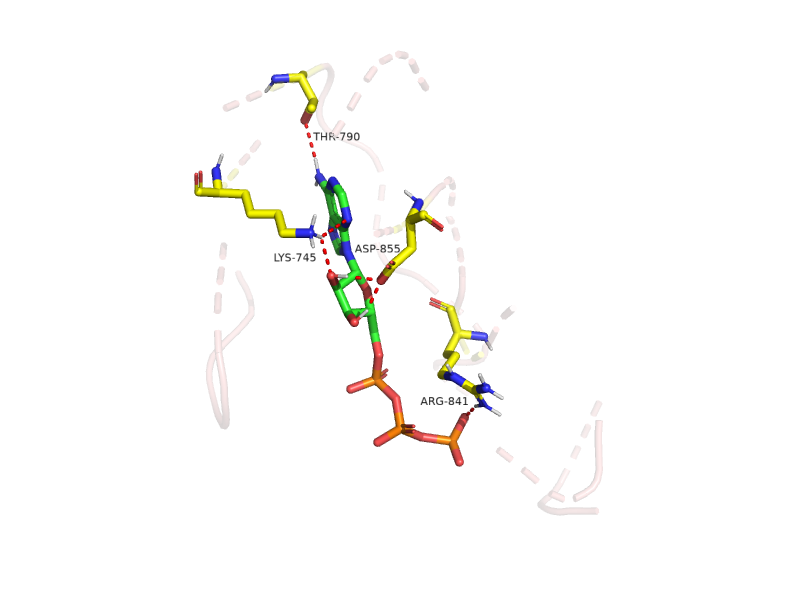


EGFR and [Adenosine Triphosphate](http://www.megabionet.org/tcmid/ingredient/626/)


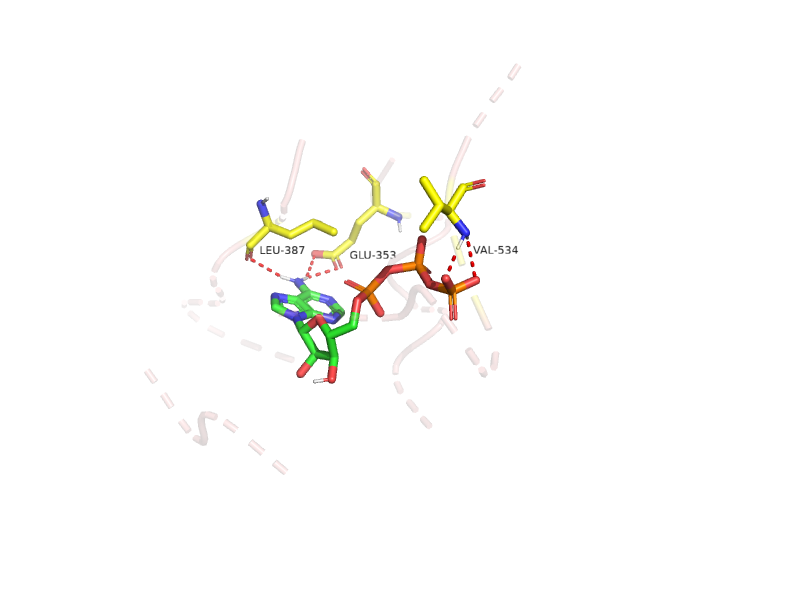

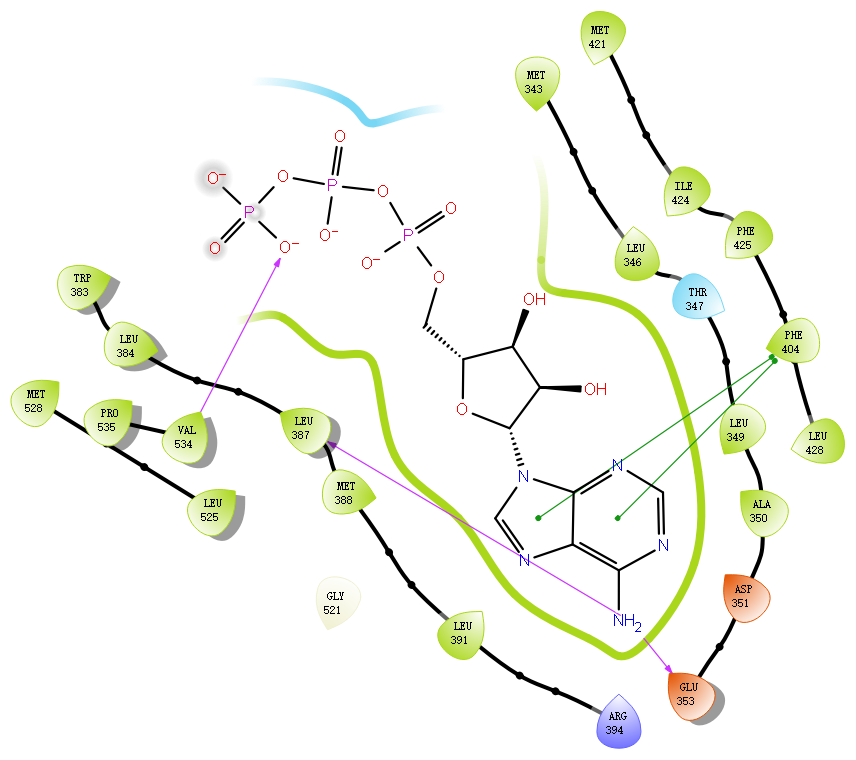


ESR1 and [Adenosine Triphosphate](http://www.megabionet.org/tcmid/ingredient/626/)


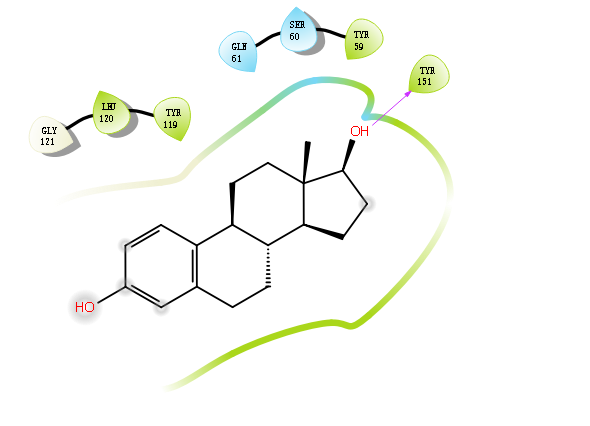

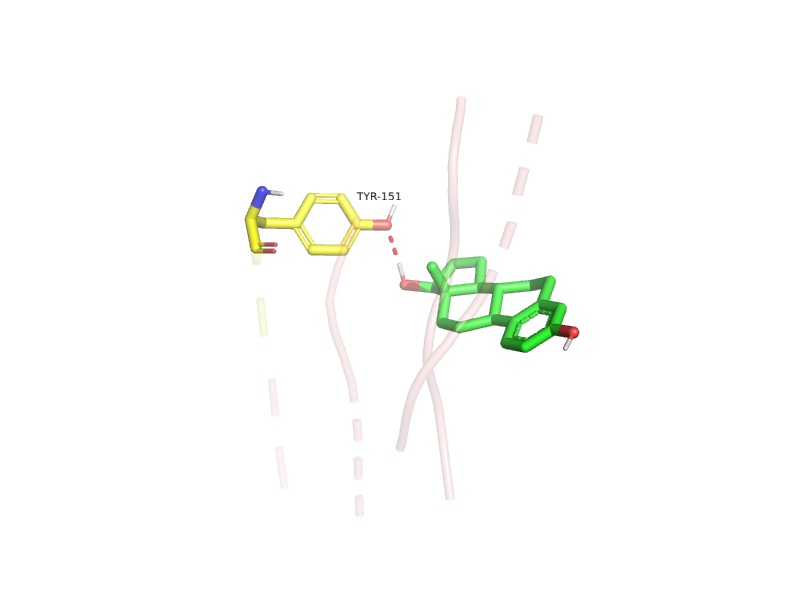


TNF and [17-Beta-Estradiol](http://www.megabionet.org/tcmid/ingredient/23197/)


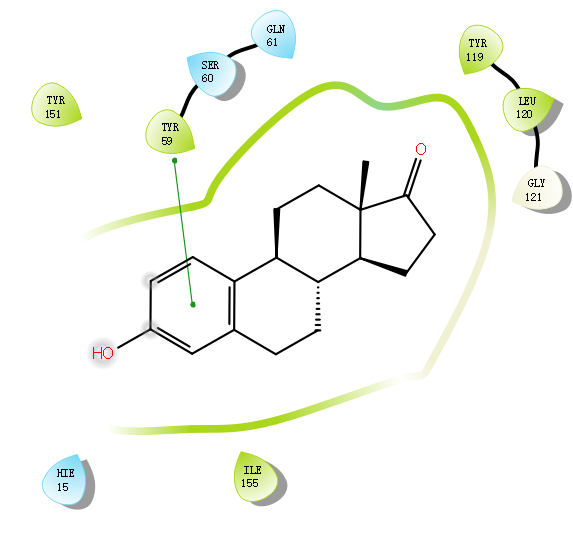


TNF and [Oestrone](http://www.megabionet.org/tcmid/ingredient/23428/)


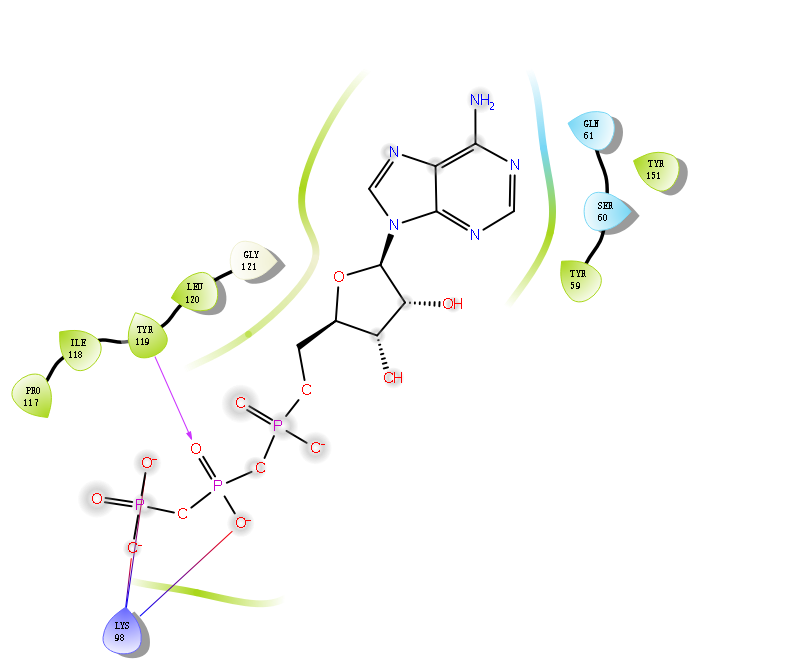

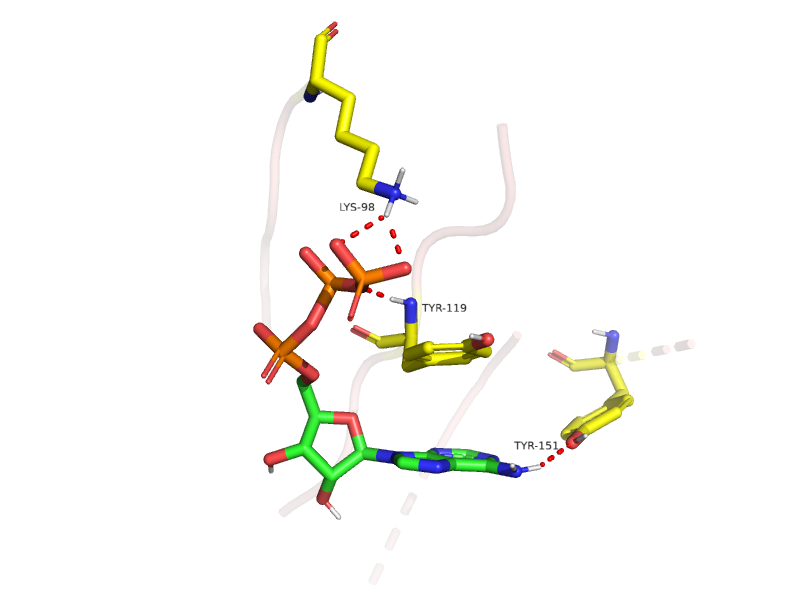


TNF and [Adenosine Triphosphate](http://www.megabionet.org/tcmid/ingredient/626/)
